# Reduction of radiation dose using real‐time visual feedback dosimetry during angiographic interventions

**DOI:** 10.1002/acm2.13860

**Published:** 2022-12-24

**Authors:** Vitali Koch, Lena Marie Conrades, Leon D. Gruenewald, Katrin Eichler, Simon S. Martin, Christian Booz, Tommaso D'Angelo, Ibrahim Yel, Simon Bernatz, Scherwin Mahmoudi, Moritz H. Albrecht, Jan‐Erik Scholtz, Axel Thalhammer, Stephan Zangos, Thomas J. Vogl, Tatjana Gruber‐Rouh

**Affiliations:** ^1^ Department of Diagnostic and Interventional Radiology University Hospital Frankfurt Frankfurt am Main Germany; ^2^ Department of Biomedical Sciences and Morphological and Functional Imaging University Hospital Messina Messina Italy

**Keywords:** angiography, in vivo dosimetry, radiation dosimetry, radiation dosage, radiation protection

## Abstract

This prospective study sought to evaluate potential savings of radiation dose to medical staff using real‐time dosimetry coupled with visual radiation dose feedback during angiographic interventions. For this purpose, we analyzed a total of 214 angiographic examinations that consisted of chemoembolizations and several other types of therapeutic interventions. The Unfors RaySafe i2 dosimeter was worn by the interventionalist at chest height over the lead protection. A total of 110 interventions were performed with real‐time radiation dosimetry allowing the interventionalist to react upon higher x‐ray exposure and 104 examinations served as the comparative group without real‐time radiation monitoring. By using the real‐time display during interventions, the overall mean operator radiation dose decreased from 3.67 (IQR, 0.95–23.01) to 2.36 μSv (IQR, 0.52–12.66) (−36%; *p* = 0.032) at simultaneously reduced operator exposure time by 4.5 min (*p* = 0.071). Dividing interventions into chemoembolizations and other types of therapeutic interventions, radiation dose decreased from 1.31 (IQR, 0.46‐3.62) to 0.95 μSv (IQR, 0.53‐3.11) and from 24.39 (IQR, 12.14‐63.0) to 10.37 μSv (IQR, 0.85‐36.84), respectively, using live‐screen dosimetry (*p* ≤ 0.005). Radiation dose reductions were also observed for the participating assistants, indicating that they could also benefit from real‐time visual feedback dosimetry during interventions (−30%; *p* = 0.039). Integration of real‐time dosimetry into clinical processes might be useful in reducing occupational radiation exposure time during angiographic interventions. The real‐time visual feedback raised the awareness of interventionalists and their assistants to the potential danger of prolonged radiation exposure leading to the adoption of radiation‐sparing practices. Therefore, it might create a safer environment for the medical staff by keeping the applied radiation exposure as low as possible.

## 1 INTRODUCTION

The last decades have brought a multitude of technological advances in radiologic imaging techniques, combined with increased accessibility and utilization. However, occupational exposure to radiation in fluoroscopy‐guided interventions remains a major concern. Especially, during complex interventional procedures, which require the radiologists to be near the patient, medical staff is frequently subject to high levels of radiation exposure.^1^ According to the new guidelines of the European Atomic Energy Community (EURATOM, directive 2013/59/Euratom),^2^ the dose limit for occupational exposure of eye lenses has been reduced from 150  mSv/year to 20  mSv/year. Considering the increased risk of medical staff to develop cancer or even deterministic skin injuries, effective strategies are needed to monitor and reduce radiation dose.^3^, ^4^

In this context, miscellaneous techniques have been proposed that aim at optimizing device settings and proper positioning of the x‐ray source.^5^, ^6^, ^7^, ^8^ The radiation dose of medical staff exposed to ionizing radiation is commonly monitored by using thermoluminescent dosimeters. However, the main drawback of this technique is that the recorded data is not available immediately after or even during the examinations. Consequently, situations with increased radiation exposure might not be associated with the causative examination, thereby hindering critical reflection and optimization of future investigations.^9^ Real‐time dosimetry can provide immediate information about occupational radiation dose rates with the opportunity to react during interventions, for example by changing the position or increasing the distance to the x‐ray source. It might raise the awareness of elevated radiation exposure according to the ALARA (As Low As Reasonably Achievable) principle which proposes to use the lowest dose possible for sufficient image quality and diagnostic information.^10^

Despite several studies in the field of occupational live‐screen dosimetry and reports on their clinical advantages,^11^, ^12^, ^13^, ^14^, ^15^ comprehensive systematic evaluations of radiation dose monitoring during angiographic interventions are sparse.

This study sought to evaluate whether digital dosimetry that offers visual real‐time feedback can reduce occupational radiation exposure to operators and their assistants during angiographic interventions.

## 2 METHODS

The present prospective study was approved by the institutional ethical review board and performed at the Department of Diagnostic and Interventional Radiology of the University Hospital Frankfurt (Frankfurt am Main, Germany).

### 2.1 Study protocol

**FIGURE 1:**
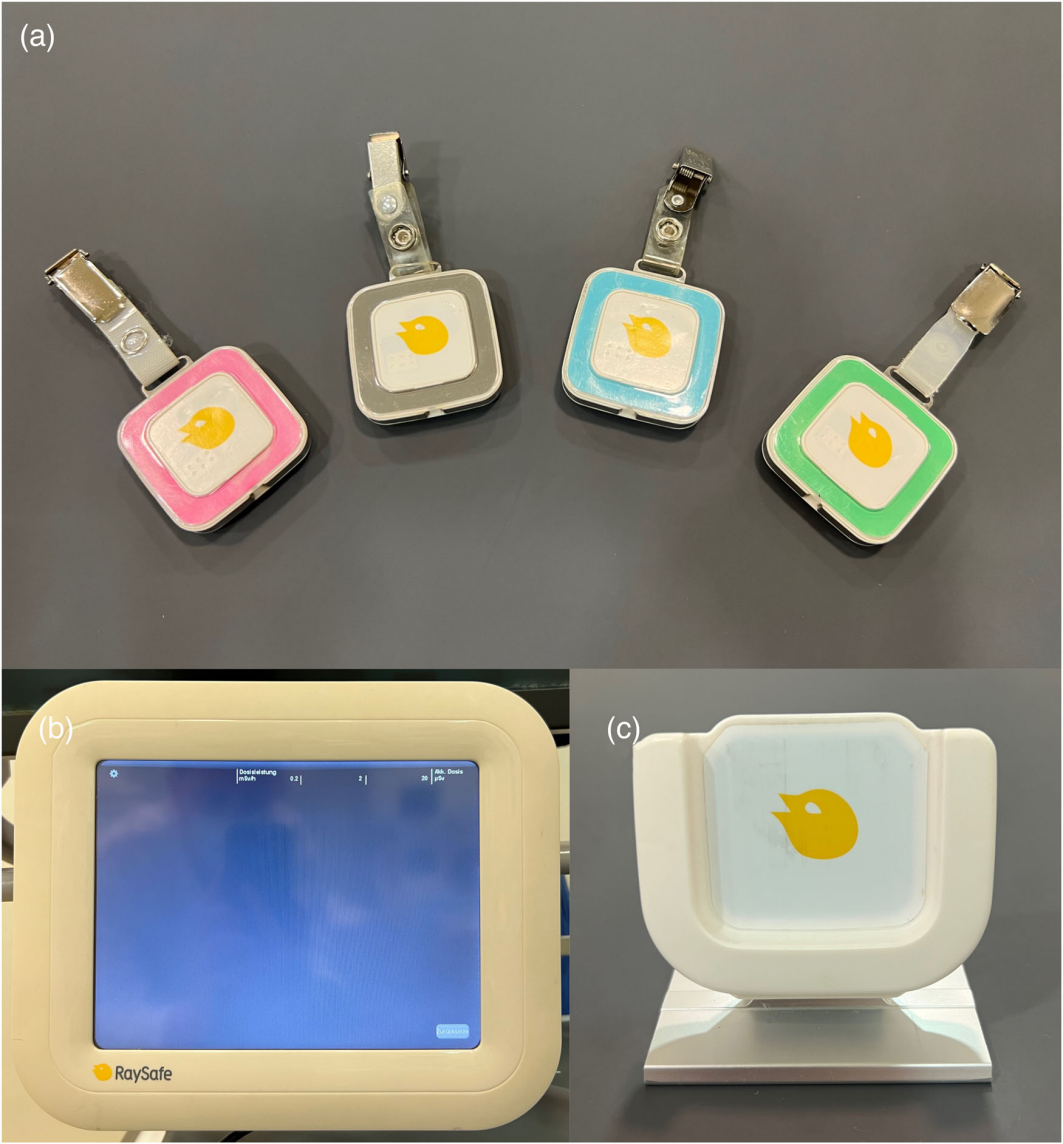
Measurement of radiation exposure (mSv/h) using a direct dosimeter system (Unfors RaySafe GmbH, Ulm, Germany) with real‐time visual feedback, consisting of dosimeters in different colors (A), a real‐time display (B), and a cradle (C). Abbreviations: mSv, millisievert. μSv, mikrosievert.

**FIGURE 2:**
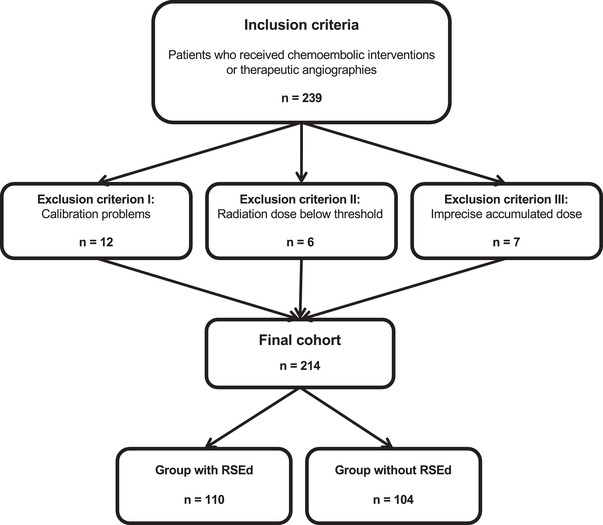
Illustration of the study design. Abbreviations: RSEd, real‐time display.

Radiation exposure was measured using the direct dosimeter system Unfors RaySafe i2 (Unfors RaySafe GmbH, Ulm, Germany), consisting of Unfors RaySafe i2 dosimeters (RSD), the Unfors RaySafe i2 real‐time display (RSEd) with a screen size of 10.4″, the Unfors RaySafe i2 dose manager 1.0.11.0 (RSDM), and the Unfors RaySafe i2 cradle (Figure 1). Measurements were recorded over a period of 1 year and contained 239 consecutive angiographic interventions. Of these, 25 angiographic examinations (11%) had to be excluded due to calibration problems (incomplete and/or imprecise calibration; *n* = 12), imprecise accumulated dose values (*n* = 7), or insufficient radiation dose to exceed the required threshold values of the RSDs (*n* = 6). Angiographic interventions of adult patients were either performed by a consultant (J.‐E.S., 6 years of experience in angiographic interventions) or two senior physicians (S.Z. and T.G.R., 18 and 16 years of experience in angiographic interventions, respectively), which were assisted by one resident physician. The angiography device was an Axiom Artis dTA from Siemens (Siemens Healthineers, Forchheim, Germany), offering a digital flat panel detector system with an image matrix of 1024^2^ pixels. All registered examinations were read out monthly assigning every single radiation measurement to the respective interventionalist and examination. The study inclusion process is illustrated in Figure 2.

For randomization, cards were labelled with 0 (examination without live‐screen dosimetry) or 1 (examination with live‐screen dosimetry) and then shuffled, allocating a distinct number to every single angiographic intervention. In some procedures, one dosimeter was given to the interventionalist and an additional dosimeter was given to the assistant. Subsequently, all 214 angiographic examinations could be divided into a group with RSEd (*n* = 110) and a group without RSEd (*n* = 104), which resulted in 110 versus 104 measurements for primary operators (∑ = 214), and 73 versus 73 measurements for assistants (∑ = 146). In the group without RSEd, there was no possibility of radiation dose control during the intervention. In the group with RSEd, the radiation exposure of the employees was displayed directly without time delay on the RSEd during the examination. Thus, it was not only possible to react to high‐exposure events in real‐time but also to allocate every single radiation exposure peak to a certain time point during the intervention. However, changes in technical parameters or the positioning of the C‐arm system were not directly linked to radiation dose recordings, requiring manual reporting and analysis. The RSEd has been placed either directly at the intervention table (*n* = 68) or at a distance of 2 m to the examiner on the wall (*n* = 57) to assess the effects of its localization at a larger distance to the investigator. To differentiate training‐related changes in behaviour from changes that might be mainly attributable to the use of live‐screen dosimetry, a total of 30 interventions that were performed with and without initial training regarding radiation dose savings and correct behaviour were additionally analyzed. The training was achieved by refreshing useful methods that aim at keeping radiation exposure as low as reasonably achievable. For this purpose, an experienced senior interventionalist with 19 years of experience in interventions (K.E.) gave clear instructions in radiation dose protection to primary investigators, each of them completing a 30‐min refresher course. The lecture included the use of table‐side shields (equipment‐related variables), keeping sufficient distance to the x‐ray tube (operator‐related variables), and technical parameters such as the adjustment of radiation input dose for heavier patients (device‐related variables). We included one group which was not initially trained on dose reduction techniques and was given the RSD and RSEd (*n* = 10), another group that was trained on dose reduction techniques and was given the RSD and RSEd (*n* = 10), and a third group that was trained on dose reduction techniques and was given the RSD but was not given the RSEd (*n* = 10).

For better comparability, groups with and without RSEd were subdivided into therapeutic angiographies (e. g., angiographies of pelvis/leg, abdomen, head/neck, transjugular intrahepatic portosystemic shunts, carbon dioxide angiographies, lipiodol angiographies, and percutaneous transluminal angioplasties) and chemoembolic interventions (comprising transarterial chemoembolizations of the liver, thoracal/abdominal, pelvis, and transarterial chemoperfusions). During the test series, separate data sets were collected for the treating radiologist and the assistant.

**FIGURE 3:**
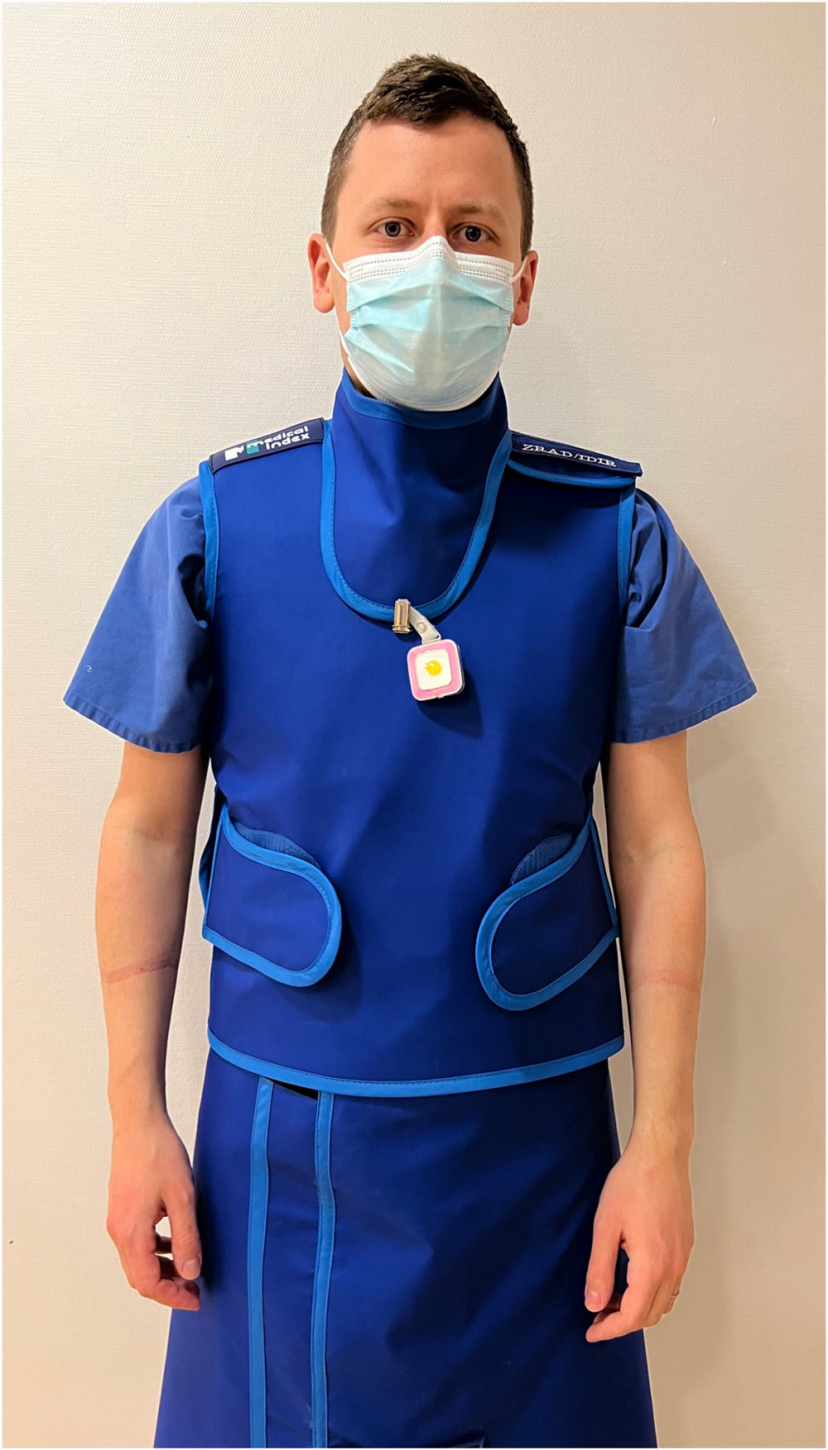
The dosimeter was worn by the interventionalist at chest height over the lead protection, communicating wirelessly with the real‐time display via a radio link. Communication was secured up to a distance of 10 m.

**FIGURE 4:**
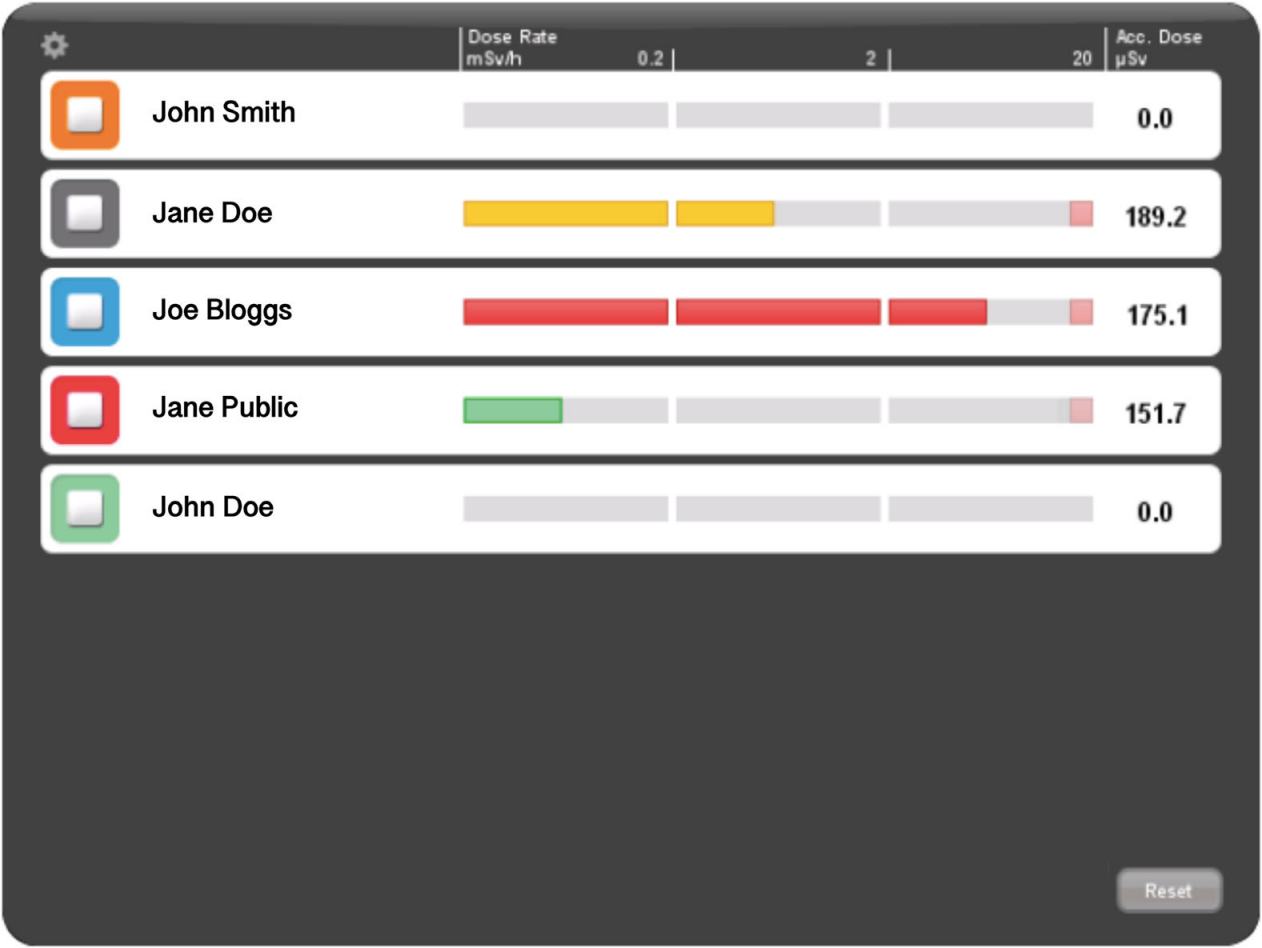
Screenshot of the RaySafe real‐time display visualizing the accumulated radiation dose using color‐coded bar graphs. Green, yellow, and red bars indicate the dose rate for the five participating individuals. Abbreviations: Acc. Dose, accumulated dose. mSv, millisievert. μSv, mikrosievert.

**FIGURE 5:**
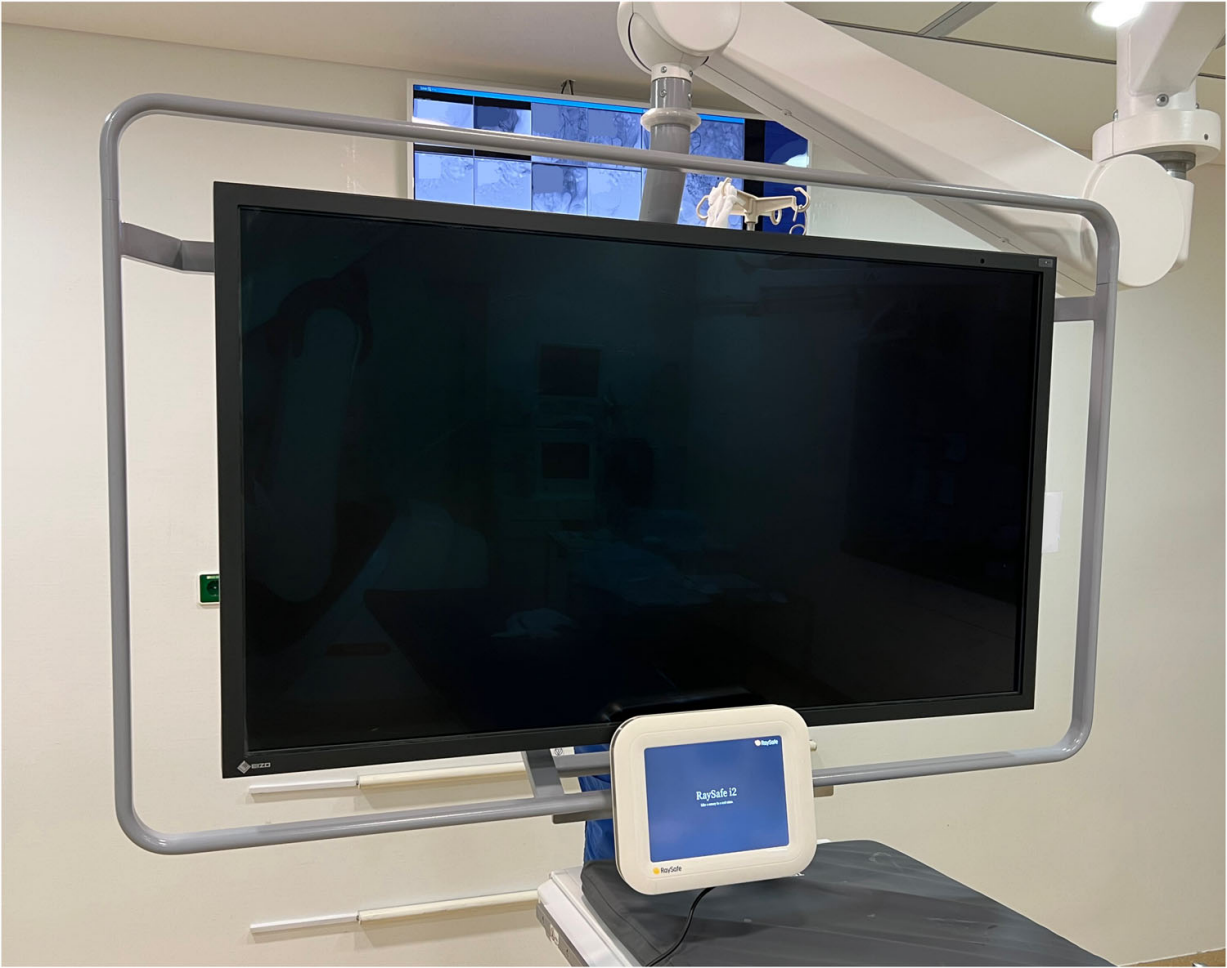
The real‐time display was attached to a height‐adjustable rod, which in turn could be mounted on the angiography table via a rail system.

The RSD was worn by the interventionalist at chest height over the lead protection (Figure 3) and communicated wirelessly with the RSEd via a radio link. Communication was secured up to a distance of 10 m. The RSEd displayed the current dose rate (mSv/h) on a bar graph. The bar was depicted in different defined colors depending on the intensity of the radiation exposure: green at values <0.2 mSv/h, yellow in the range of 0.2‐2 mSv/h, and red in the range of 2–20 mSv/h. The colors were predefined by the manufacturer, not changeable, and constructed for the purpose to offer a quick visual illustration of the current radiation dose exposure to the operator. The accumulated radiation dose using color‐coded bar graphs is illustrated in Figure 4. In the case of a visual warning of high‐radiation exposure, the operator had multiple options to reduce his radiation dose, including adjusting of shielding barriers, increasing the distance to the radiation source by changing his position, moving the image intensifier closer to the patient, shortening of the interventional time, or changing technical parameters (reduction of frame rate, energy per frame, or optimized collimation). The detection range of the RSDs was between 40 μSv/h and 300 mSv/h. There were two dose memory options, one for the accumulated dose (μSv) over the entire lifetime of the RSD, and the other for single measurements exceeding the start trigger level >40 μSv/h. The accumulated dose (μSv) was illustrated on the right‐hand side of the touch panel. In the lower right area of the RSEd was a “reset” button, which could reset the accumulated dose. In this study, the displayed radiation dose of the RSEd was reset after each intervention. The user had the option of accessing his data via the RSEd. It was possible to view the dose history as a bar graph, the annual dose from January 1st of each year, as well as individual measurements. All recorded data could be downloaded for further evaluation on a personal computer by applying the RSDM software. Using the Vogel's WALL 1005 mount (Vogel's Deutschland GmbH & Co. KG, Löhne, Germany), the RSEd was attached to a height‐adjustable rod, which in turn could be mounted on the angiography table via a rail system (Figure 5).

The Unfors RaySafe i2 cradle automatically served as a charging station for the RSD as long as it was connected to the computer. The battery consumption of the RSD was low and did not result in any failures in this study. The RSDM software was used for dose evaluation and settings of the RSDs. The home screen allowed a rapid overview of all dosimeters currently in use providing the ID and name of the RSD, the accumulated dose, the remaining memory place, and the battery status.

The date and operator exposure time of the intervention, the wearer of the dosimeter, and the type of intervention were recorded together with the information on whether the RSEd was used. During all interventions, standard radiation protection devices were used, such as lead rubber‐filled strips and lead glass planes.

To better understand the preferences of operators and assistants and the value of real‐time dosimetry in the context of various other radiation protection measures, we performed a small‐scale survey involving senior interventionalists (*n* = 8) and assisting residents (*n* = 10). The medical staff was asked to rate the following radiation protection measures: ‘radiation‐free alternatives’, ‘regular education and training’, ‘limited length of stay for ancillary staff’, ‘personal protection clothing’, ‘mobile shielding’, ‘table‐based shielding’, ‘distance to the x‐ray tube’, ‘examination time’, ‘pulse rate lowering’, ‘filtering and collimation’, ‘automatic tube voltage modulation’, ‘low‐dose protocol’, ‘c‐arm angulation’, ‘real‐time dosimetry’, and ‘conventional dosimetry’. All items were rated on a 5‐point Likert scale (range, 1–5), with 1 indicating ‘agree very strongly’ to 5 ‘disagree very strongly’.

### 2.2 Statistical analysis

Statistical analysis was performed using the software package from MedCalc (MedCalc, Version 20.022, Ostend, Belgium). Normal distribution was tested by applying the Shapiro‐Wilk test. Continuous variables were reported as mean ± standard deviation (SD), or as median with interquartile range (IQR). Categorical variables were expressed as numbers with their corresponding percentage. Comparisons between operator radiation doses with or without real‐time monitoring were performed using one‐way ANOVA, Mann‐Whitney test, or two‐tailed Student's t‐test, where appropriate. Ratings from the survey analysis were compared using the Mann‐Whitney test. A two‐sided *p* value of <0.05 was considered indicative of a statistically significant difference.

## 3 RESULTS

From a total of 388 measurements, 93% were finally evaluated, which corresponds to 360 measurements for operators and their assistants.

Overall operator radiation doses ranged from 0.05 to 161.03 μSv (median, 2.36 μSv, IQR 0.52–12.66) in the group with RSEd and from 0.03 to 474.73 μSv (median, 3.67 μSv, IQR 0.95–23.01) in the group without RSEd (−36%; *p* = 0.032). Radiation doses scattered particularly in the upper dose ranges which can be explained by sometimes challenging interventional procedures (for example due to more complex anatomical conditions).

For chemoembolizations, measured operator dose values ranged from 0.05 to 34.94 μSv (median, 0.95 μSv, IQR 0.53–3.11) in examinations with RSEd and from 0.03 to 81.46 μSv (median, 1.31 μSv, IQR 0.46–3.62) in those without using the RSEd (−28%; *p* = 0.004).

For therapeutic angiographies, examinations with RSEd (median, 10.37 μSv, IQR 0.85–36.84) differed significantly from those without RSEd (median, 24.39 μSv, IQR 12.14–63.0) (−58%; *p* = 0.005). In comparison to chemoembolizations, the operator dose values scattered widely from 0.08 μSv in examinations with RSEd to over 400 μSv in those without RSEd, mostly due to elaborate techniques, difficult conditions or complex anatomy. Measured radiation doses are summarized in Table 1.

**TABLE 1:** Overview of operator radiation doses (μSv) at chemoembolizations and other types of angiographic interventions

	Operator radiation dose without RSEd [μSv]	Operator radiation dose with RSEd [μSv]	*p*‐value
Different types of therapeutic angiographies other than chemoembolizations (median, IQR)	24.39 (12.14–63.0)	10.37 (0.85–36.84)	0.005
Chemoembolizations (median, IQR)	1.31 (0.46–3.62)	0.95 (0.53–3.11)	0.004
Overall (median, IQR)	3.67 (0.95–23.01)	2.36 (0.52–12.66)	0.032

Abbreviations: RSEd, real‐time display. IQR, interquartile range. μSv, mikrosievert.

Operator radiation rate (mSv/h) and operator exposure time did not differ between examinations with and without RSEd, showing a reduction of 16% and 4.5 min on average (*p* = 0.24 and *p* = 0.071, respectively). In general, radiation doses measured at chemoembolizations were lower compared to those obtained for other therapeutic angiographies (*p* < 0.05).

The dose values of the operators were compared depending on the position of the RSEd which has been placed either directly at the intervention table or at a distance of 2 meters on the wall. We observed a dose reduction of 3% for the 68 measurements when RSEds were placed at the intervention table in front of the interventionalist compared to the 57 measurements recorded at wall positioning (mean, 15.57 vs. 16.06 μSv; *p* = 0.05).

To differentiate training‐related behavioural changes from changes caused by the use of live‐screen dosimetry, we analyzed 30 interventions that were performed with and without initial training regarding radiation dose savings and correct behaviour. The addition of initial training to the use of the RSD and RSEd did not lead to significant reductions in radiation dose compared to live‐screen dosimetry alone (median, 18.76 μSv, IQR 12.87–25.83 vs. 30.03 μSv, IQR 15.87–38.94; *p* = 0.094). The subgroup with training and RSD but not RSEd (median, 48.59 μSv, IQR 34.79–66.23) differed significantly from the training and non‐training groups using the visualization monitor (*p* = 0.001 and *p* = 0.043, respectively).

Overall radiation dose values (irrespective of using the RSEd) of assistants were compared to those of accompanying primary operators, revealing no statistically significant differences (mean, 29.66 vs. 28.75 μSv, *p* = 0.89). After dividing assistants into subgroups with and without live‐screen dosimetry, radiation dose values differed between assistants using real‐time dosimetry (median, 8.67 μSv, IQR 3.87–14.51) and those without (median, 12.33 μSv, IQR 7.02–21.42) (−30%; *p* = 0.039).

We have also examined whether interventional experience and the level of training had an impact on radiation exposure. For this purpose, two senior physicians with profound interventional skills were selected and radiation dose exposure was measured with and without live‐screen dosimetry. We observed a radiation dose reduction by 63% using the RSEd (mean, 17.95 vs. 47.96 μSv; *p* = 0.0002) despite frequently long intervention times.

In the questionnaire analysis on the preferences and value of miscellaneous radiation protection measures (Table 2), ratings did not differ between senior interventionalists and assisting residents (*p* ≥ 0.321).

**TABLE 2:** Results of the survey involving senior interventionalists (*n* = 8) and assisting residents (*n* = 10) covering a comprehensive set of points for radiation‐sparing practices

Survey results score (1–5) ‐ median (interquartile range)	Senior physicians (*n* = 8)	Assisting residents (*n* = 10)	*p*‐value
** General management**			
Radiation‐free alternatives	1.5 (1.0–2.0)	2.0 (1.0–2.0)	0.8835
Regular education and training	1.5 (1.0–2.0)	1.0 (1.0–1.3)	0.3213
Limited length of stay for ancillary staff	1.0 (1.0–1.8)	1.0 (1.0–1.0)	0.4444
** Equipment‐related variables**			
Personal protection clothing	1.0 (1.0–1.0)	1.0 (1.0–1.0)	0.9999
Mobile shielding	2.0 (1.0–2.0)	1.0 (1.0–2.0)	0.4558
Table‐based shielding	1.0 (1.0–2.0)	1.0 (1.0–1.3)	0.6078
** Operator‐related variables**			
Distance to the x‐ray tube	1.0 (1.0–1.0)	1.0 (1.0–1.0)	0.9999
Examination time	1.0 (1.0–1.8)	1.0 (1.0–1.3)	0.9999
** Device‐related variables**			
Pulse rate lowering	1.0 (1.0–2.0)	1.5 (1.0–2.0)	0.5431
Filtering and collimation	1.0 (1.0–2.0)	1.0 (1.0–2.0)	0.9999
Automatic tube voltage modulation	1.5 (1.0–2.0)	1.0 (1.0–2.0)	0.6305
Low‐dose protocol	2.0 (1.0–2.0)	1.5 (1.0–2.0)	0.7044
C‐arm angulation	1.5 (1.0–2.0)	1.0 (1.0–2.0	0.9999
** Dosimetry**			
Real‐time dosimetry	1.0 (1.0–2.0)	1.0 (1.0–1.3)	0.6078
Conventional dosimetry	2.0 (1.3–2.0)	2.0 (1.0–3.0)	0.9232

## 4 DISCUSSION

This study sought to investigate whether real‐time radiation monitoring has the potential to save radiation dose by offering immediate visual feedback during angiographic interventions. We found overall reductions in operator radiation dose by 36% (*p* = 0.032) and operator exposure time by 4.5 min (*p* = 0.071) when real‐time dosimetry was used. It is important to note that both factors stand in a direct relationship since increased awareness of radiation exposure usually leads to reduced operator exposure times that automatically lower radiation doses for the interventionalist acting in front of the x‐ray source as well as the assistant behind him. Comparing interventions regarding radiation dose savings by using live‐screen radiation monitoring, we observed a significant reduction in radiation exposure for chemoembolizations (−28%, *p* = 0.004) and all other types of therapeutic interventions (−58%, *p* = 0.005).

The type of intervention plays an important role in the degree of exposure to ionizing radiation.^16^ Some interventions are technically more complex due to individual anatomy (e.g., pelvic leg angiographies) resulting in increased radiation exposure, whereas chemoembolizations usually have shorter intervention times.^17^, ^18^ In addition, some patients were treated repeatedly using the same intervention protocol, and the anatomy was frequently known to the radiologist from previous examinations. Nevertheless, all techniques showed a significant reduction in radiation exposure when using live‐screen dosimetry. A clear difference is particularly notable for pelvic/leg angiography, in which the digital subtraction angiography (DSA) technique was applied, showing a reduction of radiation dose by 44% in comparison to patients undergoing transarterial chemoembolizations with a radiation dose reduction of 16% on average.

Undoubtedly, the knowledge and experience level of the performing interventionalist may also be relevant for the final extent of radiation exposure.^19^ Based on our experience in clinical practices, it can also be difficult for senior radiologists with advanced skills to consistently maintain low exposure to ionizing radiation. Nevertheless, experienced senior physicians might also benefit from live‐screen dosimetry reducing their radiation exposure to lower levels even in case of complex interventions. Here we show that, despite sometimes long intervention times, there is still a significant reduction in radiation exposure, positively influencing the x‐ray behaviour of experienced interventional radiologists.

In this context, the question of what exactly has led to radiation dose reductions is very interesting and deserves further investigation. Especially the impact of dedicated instructions and training for saving radiation dose before initiating an angiographic intervention is of interest to differentiate training‐related changes in behaviour from changes that might be attributable to the use of the live‐screen dosimeter. With proper training, interventionalists can be taught how to yield similar image projections with similar outcomes at reduced radiation dose exposure. Therefore, we initiated another small investigation including a total of 30 interventions that were performed with and without initial training regarding radiation dose savings. Interestingly, the addition of initial training to the use of the real‐time radiation device did not lead to significant reductions in radiation dose compared with live‐screen dosimetry alone (*p* = 0.094). However, the subgroup with training and RSD but not RSEd differed significantly from the training and non‐training groups using the visualization monitor (*p* = 0.001 and *p* = 0.043, respetively). This indicates that not necessarily the training of interventionalists or the awareness of wearing a real‐time dosimeter might be responsible for radiation dose savings, but rather the continuous visual feedback of the current radiation dose exposure.

Interestingly, no dose reduction was observed between interventionalists and their assistants. A possible explanation would be that the assistant was not able to shield himself sufficiently from scattered radiation despite the greater distance to the x‐ray tube, while the primary investigator could individually adjust all available protective equipment. To better understand what kind of safeguards or changes in behaviour are effective in reducing the radiation dose of operators and ancillary staff, a survey has been performed that involved both senior interventionalists (*n* = 8) and assisting residents (*n* = 10). Recommendations included positioning beyond the radiation field aside from the primary investigator, using additional protective equipment like mobile table‐side shields, or immediately leaving the examination after successful assistance during the main procedure. Interestingly, ratings did not differ between senior interventionalists and their assisting residents (*p* ≥ 0.321), reaching high values for the majority of radiation protection measures including real‐time dosimetry. It demonstrates that there isn't much of a perceived preference for the various methods of dose reduction. Thoughtful radiation management should take several factors into account and not be debated as an isolated issue solely focusing on the right use of protective equipment. In our daily clinical experience, we observed an increased awareness of the assistant to a higher radiation dose exposure if the radiation dose monitor was positioned directly in front of both investigators, or when the primary investigator stopped to adjust either protective equipment or his position aiming at reducing the displayed radiation dose. In this context, radiation dose values differed between assistants using real‐time dosimetry and those without live‐screen dosimeters (−30%; *p* = 0.039). Therefore, participating assistants could also benefit from real‐time visual feedback during interventions. However, larger screens for real‐time radiation dose monitoring and extended options for the protection of ancillary staff are necessary to ensure a more effective radioprotection, especially in the case of more than one participating assistant. Furthermore, ancillary staff should receive thorough instructions and appropriate training before assisting in all kinds of angiographic examinations. Sufficient knowledge about occupational radiation exposure remains a crucial issue for both interventionalists and their assistants to increase their awareness and promote the application of additional protective measures and shielding barriers. Future studies are needed to assess potential radiation dose savings using live‐screen dosimetry if more than one assistant participates.

The localization of the RSEd also seems to play a role. If the device was not directly installed in the radiologist's field of view, the dose reduction was less pronounced than with live‐screen dosimetry directly attached to the intervention table (*p* = 0.05). Due to the small screen size of the RSEd (10.4″), it was sometimes difficult to recognize changes in radiation exposure from a larger distance to the examiner. For this reason, we recommend its positioning close to the radiologist or the use of correspondingly larger screens.

Technical innovations and advances in hard‐ and software have stimulated a multitude of different designs of radiation monitoring systems with the option to measure radiation dose in real‐time, receiving auditory or visual feedback. Considering an aging population, this process is expected to even increase in the future. The investigated real‐time dosimetry system was specifically designed to record and monitor the radiation exposure of medical staff via a real‐time display of the current radiation dose during miscellaneous radiological procedures. It represented merely an addition to the required conventional dosimetry devices with thermoluminescence technique, not intended to replace them. In our study, the trigger level of the Unfors RaySafe i2 dosimeters to start radiation dose measurement was between 40 μSv/h and 300 mSv/h. If the dose has dropped below a level of 40 μSv/h, the measurement stopped until the radiation exposure again exceeded the start trigger level. The system operated with similar dose rejection thresholds as live‐screen dosimeter systems from other manufacturers,^9^, ^20^, ^21^, ^22^, ^23^, ^24^ which increases the comparability of measurements. Furthermore, we believe that the performance in other institutions with similarly organized angiographic structures will not differ significantly given the reproducible results of radiation dose measurements in our study.

Despite simple handling and good real‐time visualization due to color‐coded bars, only 93% of measured interventions could be evaluated. In the case of the remaining 7%, the RSDs did not recognize the investigation because of a too‐low radiation dose or provided only imprecise data if the capacity of the time memory was exceeded. In such cases, the dose was displayed as an hourly dose value and not considered if more than one intervention took place in 1 h. Additionally, some measurements showed time differences that avoided the right assignment of measurements despite regular time calibration via the RSDM. The reasons for this could be the projection angle with varying amounts of radiation dose or a too‐short beam time.

From our experience in daily clinical processes, the dosimeter can be classified as user‐friendly in visualizing radiation doses for interventionalists and self‐monitoring by giving an optical warning whenever the radiation dose is too high. Increasing awareness and sensitization to long‐term damages of ionizing radiation can promote dealing with radiation protection measures and change x‐ray behaviour. By getting used to a permanent risk due to insufficient awareness, the radiation during radiological interventions may be underestimated and not perceived as a potential danger due to the lack of immediate direct radiation effects. Therefore, more experienced radiologists might wear the RSD at regular intervals over longer periods to make them aware of the dangers to which they are exposed daily. It is also possible to use the RSD system as a personal dosimeter, enabling direct evaluation of individual accumulated radiation doses to increase the awareness of long‐term damages. This might implicate not only a significant exposure reduction for the radiologist himself but also the patient.^25^, ^26^

In terms of radiation dose savings, several studies reported similar reductions in radiation dose when using live‐screen dosimetry.^9^, ^21^, ^22^, ^23^ Khan et al. found a decreased radiation dose of up to 36% and reduced mean operator exposure times using a real‐time dosimeter with auditory feedback.^22^ In light of the increasing number of treatments in interventional radiology due to its minimal invasiveness, radiation management should be an integral part of every radiological department to closely monitor interventions. The application of the real‐time dosimetry system has led to several changes during angiographic procedures in our department. To further reduce the exposure to ionizing radiation, it is now possible to leave the room at each angiography for contrast medium injection (e.g., during the DSA series) and to control the process from outside by using a manual switch.

Several limitations have to be addressed. First, this was a prospective study with a limited number of measurements requiring further validation. Second, the recorded data had to be extracted from the RSEd via a universal serial bus (USB) interface for further evaluation on a personal computer. A wireless connection to a mobile device with a dedicated app would be desirable enabling the possibility to immediately access and evaluate all recorded data. Third, the real‐time dosimetry system in our study was intended to be used as a permanently installed system, requiring a continuous power supply. Integrating a rechargeable battery would markedly increase its flexibility and facilitate radiation dose monitoring in different rooms. Fourth, a correlation between the radiation exposure measured by our device and the recordings from conventional thermoluminescent dosimeter systems continuously worn by the entire medical staff could not be evaluated. Fifth, the detection range of RSDs ranged between 40 μSv/h and 300 mSv/h, which prohibited the inclusion of investigations below this threshold. Finally, our study findings may not be transferable to devices from other manufacturers and may not be generalizable to investigations in other fields of radiation exposure.

## 5 CONCLUSION

In the context of previous studies,^9^, ^21^, ^22^, ^23^ the real‐time dosimetry system in our study can be recommended as an integral part of a radiologist's training program, especially for young radiologists as a personal dosimeter. Since people exposed to radiation are at increased risk to develop cancer due to stochastic radiation effects, this type of training in connection with the given radiation protection devices is urgently needed to limit the exposure and thus a possible increased risk of illness to a minimum.

The evaluated direct dosimeter can be regarded as a useful addition to radiation protection. Due to easy handling and high user‐friendliness, it is versatile to use and does not require time‐consuming training to explore the user interface. The instant feedback, when exposed to high radiation, empowers interventionalists to take immediate action and minimize unnecessary radiation exposure. Therefore, real‐time dosimetry might not only positively influence young physicians starting their careers but also sensitize experienced radiologists regarding radiation exposure in their daily clinical practices.

## AUTHOR CONTRIBUTION

Vitali Koch performed evaluation of the results and wrote the manuscript. Lena Marie Conrades analyzed and interpreted the patient data. Leon D. Gruenewald, Christian Booz, Ibrahim Yel, Simon Bernatz, Scherwin Mahmoudi, Katrin Eichler and Simon S. Martin performed measurements and contributed to the illustration of figures and tables. Tommaso D'Angelo, Moritz H. Albrecht, Jan‐Erik Scholtz and Axel Thalhammer performed statistical analysis and supervised the project. Stephan Zangos, Thomas J. Vogl and Tatjana Gruber‐Rouh designed the model and provided revisions to scientific content of the manuscript. All authors have participated in this work and have reviewed and agreed with the content of the article.

## CONFLICT OF INTEREST

Ibrahim Yel received a speaking fee from Siemens Healthineers. Christian Booz received speaking fees from Siemens Healthineers. The other authors have no potential conflict of interest to disclose.
